# Understanding thrombosis with thrombocytopenia syndrome after COVID-19 vaccination

**DOI:** 10.1038/s41541-022-00569-8

**Published:** 2022-11-09

**Authors:** Alessandra Buoninfante, Arno Andeweg, Alexander T. Baker, Mitesh Borad, Nigel Crawford, Jean-Michel Dogné, David Garcia-Azorin, Andreas Greinacher, Rita Helfand, Anders Hviid, Stefan Kochanek, Marta López-Fauqued, Ishac Nazy, Anand Padmanabhan, Sue Pavord, Daniel Prieto-Alhambra, Huyen Tran, Ulla Wandel Liminga, Marco Cavaleri

**Affiliations:** 1 Health Threats and Vaccines Strategy, European Medicines Agency, Amsterdam, the Netherlands; 2 Division of Hematology and Medical Oncology, Mayo Clinic, Scottsdale, AZ 85054 USA; 3 Division of Cancer and Genetics, School of Medicine, Cardiff University, Cardiff, CF14 4XN UK; 4 Mayo Clinic Cancer Center, Phoenix, AZ 85054 USA; 5 Royal Children’s Hospital, Murdoch Children’s Research Institute, Department Paediatrics, The University of Melbourne, Melbourne, VIC Australia; 6 Department of Pharmacy, Namur Research Institute for Life Sciences, University of Namur, Namur, Belgium; 7 EMA Pharmacovigilance Risk Assessment Committee member, Amsterdam, The Netherlands; 8 Department of Neurology, Hospital Clínico Universitario de Valladolid, Valladolid, España; 9 Department of Transfusion Medicine, University Medicine Greifswald, Greifswald, Germany; 10 National Center for Emerging and Zoonotic Infectious Diseases, CDC, Atlanta, USA; 11 WHO’s Global Advisory Committee on Vaccine Safety, WHO, Geneva, Switzerland; 12 Pharmacovigilance Research Center, Department of Drug Development and Clinical Pharmacology, Faculty of Health and Medical Sciences, University of Copenhagen, Copenhagen, Denmark; 13 Department of Epidemiology Research, Statens Serum Institut, Copenhagen, Denmark; 14 Department of Gene Therapy, University of Ulm, Ulm, Germany; 15 Vaccines and Therapies for Infectious Diseases, European Medicines Agency, Amsterdam, the Netherlands; 16 McMaster Centre for Transfusion Research, McMaster University, Hamilton, ON Canada; 17 Department of Laboratory Medicine and Pathology, Mayo Clinic, Rochester, MN USA; 18 Department Hematology, Oxford University Hospitals NHS Foundation Trust, Oxfordshire, UK; 19 Centre for Statistics in Medicine (CSM), Nuffield Department of Orthopaedics, Rheumatology and Musculoskeletal Sciences (NDROMS), University of Oxford, Oxford, UK; 20 Department of Medical Informatics, Erasmus University Medical Center, Rotterdam, The Netherlands; 21 Department of Clinical Haematology, The Alfred Hospital, Melbourne, VIC Australia; 22 Australian Centre for Blood Diseases, Central Clinical School, Monash University, Melbourne, VIC Australia; 23 Medical Products Agency, Uppsala, Sweden; 24 EMA Emergency Task Force Chair, Amsterdam, The Netherlands

**Keywords:** Medical research, Vaccines

## Abstract

Safety and efficacy of vaccines against the SARS-CoV-2 coronavirus has been demonstrated in clinical trials and next by their real world use through the course of the ongoing COVID-19 pandemic. However, very rare adverse events have been detected post-authorization in certain parts of the world. This meeting report summarizes an EMA workshop’s discussion on the epidemiology, clinical presentation and biology of thrombosis with thrombocytopenia syndrome after adenovirus vector COVID-19 vaccination. General agreement was reached by international regulators, scientists and developers on the steps needed to fill the gaps in the characterization of this new syndrome. In particular, actions should be taken to improve the post-vaccination surveillance activities in low and middle income countries and investigate potential genetic predisposition factors.

Vaccines have been pivotal to prevent infectious diseases and have shown their immense public health value in reducing morbidity and mortality associated with transmissible diseases. Following the WHO declaration of the COVID-19 pandemic^1^ caused by the SARS-COV-2 virus, a global effort was made to develop vaccines against COVID-19, with a number of those being approved in expedited manner in the European Union (EU)^2^ and worldwide. Following the approval and rapid roll-out of adenovirus vector COVID-19 vaccines, very rare cases of thromboembolic events with low platelet counts were reported among people vaccinated with ChAdOx1-S AstraZeneca and Ad26.COV2.S Janssen COVID-19 vaccines, Vaxzevria and JCOVDEN, respectively, initially in Austria and Denmark^3^ and subsequently all over Europe. These cases emerged also in North America^4^ and Australia^5^. Vaccinees affected by thrombosis with thrombocytopenia manifested severe cases of thrombosis, including unusual sites such as cerebral venous sinus thrombosis, splanchnic vein thrombosis, as well as arterial thrombosis, concomitant with thrombocytopenia, and often presence of anti-platelet factor 4 (PF4) antibodies. In addition, high mortality rates in those patients were observed^6^. This syndrome named ‘thrombosis with thrombocytopenia syndrome’ (TTS) or when TTS is not better accounted for by another cause and anti-PF4 antibodies are present, is referred as vaccine-induced immune thrombotic thrombocytopenia (VITT), a newly identified very rare adverse reaction that in the majority of individuals occurs within the first three weeks following vaccination with the first dose of COVID-19 adenovirus vector vaccines. Estimations of the incidence of TTS range from 3.2 to 16.1 cases per million doses for Vaxzevria and 1.7 to 3.7 cases per million doses for JCOVDEN^7^. Reporting rates of Convidencia, an adenovirus type 5-based vector vaccine, are 0.0081 cases per million vaccinees^8^. It has been suggested that TTS/VITT occurs more frequently after the ChAdOx1-S AstraZeneca then after Ad26.COV2.S Janssen COVID-19 vaccines. However, differences in the frequency of use of these vaccines and differences in the surveillance methods across the world make it difficult to draw definite conclusions. As more cases of TTS post vaccination emerged, researchers and relevant stakeholders began to study the disease mechanism underlying this rare adverse event.

On 27 June 2022, the European Medicines Agency (EMA) hosted a virtual workshop to review the current understanding of the pathophysiology of TTS post-vaccination, to foster the discussion on potential disease mechanisms and to identify the most important next steps in the research agenda. The discussion on the benefit–risk ratio of the adenovirus vector COVID-19 vaccines, which remains positive despite the risk of TTS and VITT, was not within the remit of this workshop and is documented elsewhere^7,9–11^. Of note, the long term adverse consequences of TTS and VITT can be substantially reduced by early recognition and appropriate treatment.

## Pharmacoepidemiology: population-based studies of vaccine associated TTS

One of the aims of the workshop was to provide an overview of the population-based studies of vaccine associated TTS and to contextualize the risk and definition of TTS. The challenges and the learning opportunities in the characterisation of background rates of rare adverse events like TTS were presented. The significant level of heterogeneity related to the clinical data available as well as to the study designs applied, poses a significant challenge to define background rates accurately. As TTS post-vaccination is a newly identified disease and no standard clinical codes yet exist for it, its identification based on real-world evidence relies on the integration of (other) diagnostic codes and laboratory read-outs. The absence of anti-PF4 antibody measurements and inconsistent availability of platelet counts, depending on the clinical setting, appeared to be a major limiting factor^12^. Despite these challenges, analyses conducted by Alhambra et al.^13^ suggested an increased risk of thrombocytopenia and TTS following COVID-19 adenovirus vector compared to mRNA vaccination.

Three observational analytical approaches, i.e. observed versus expected, contemporary cohort analysis, and self-controlled case series analysis, were used in the European Nordic countries to study thromboembolic- and thrombocytopenic events after COVID-19 vaccination. Application of these methodologies suggested, collectively, that cerebral venous thrombosis (CVT) and thrombocytopenia (TP) are very rare events after Vaxzevria vaccination, 1.6–2.5 cases per 100,000 vaccinations for CVT, and 2.4–4.9 cases per 100,000 vaccinations for TP, respectively. Of note, this pattern was not observed post-vaccination with either of the two COVID-19 mRNA vaccines^14^.

At a global level, the updated WHO guidance for clinical management of TTS following COVID-19 vaccination and the journey to the agreed treatments were presented. Data of TTS reports after adenovirus vector vaccination from low and middle income countries (LMICs) were analyzed from VigiBase for recovery, death and intracranial hemorrhage. TTS cases in LMICs were associated with a mortality rate of 31.6%. In addition, a literature review was done for updating the WHO TTS guidelines. The analysis concluded that intravenous immunoglobulin and non-heparin-based anticoagulants should be used for individuals with vaccine associated TTS, and heparin may be used for anti-coagulation for individuals with TTS following vaccination when non-heparin-based anticoagulants are not available^15^. Platelet transfusion should only be used for individuals with TTS following vaccination in emergency cases where platelet transfusion is strongly indicated (i.e., active bleeding and need for urgent surgery)^16^.

The marketing authorization holders for both EU approved adenovirus vector COVID-19 vaccines shared the results of their ongoing epidemiology research and related activities to further estimate the risk of vaccine-associated TTS. The TTS reporting rates from Janssen’s Global Safety Database and characteristics of JCOVDEN vaccinees being followed for adverse events in rapid cycle analysis of real-world US healthcare administrative claims data sources were showed. It was concluded that the limited availability of relevant clinical and laboratory data hampers the case ascertainment and more specific algorithms are required to identify vaccine associated TTS in real-world data (RWD). According to the company, without information on D-dimer levels, which is linked to blood clots, anti-PF4 antibodies and platelet counts, algorithms developed to identify events based on the existing case definitions will only be able to measure the incidence of simultaneous occurring thrombosis and thrombocytopenia rather than true vaccine associated TTS rate in RWD. Janssen proposed to improve the process of case identification and implement it in the ongoing post-authorisation safety studies for JCOVDEN.

On the basis of AstraZeneca safety database analyses the key messages were that consensus on the definitions of TTS and VITT is needed to enable consistent analysis and interpretation of existing and emerging data. Despite the current analysis limitations, reporting rates seem to vary across geographical areas^17^.

The discussion focused on the main challenges associated with TTS signal detection, case definition, and the limited availability of electronic medical records, and laboratory data, particularly in remote places in low- and middle-income countries and therefore the need for a better infrastructure and (global) collaboration was widely acknowledged. Despite these limitations it was overall recognized that vaccine associated TTS reporting differs significantly from country to country. In addition to the infrastructural challenges especially in certain locations, it could not be excluded that differences in reporting rate may also originate from ethnicity and/or genetic factors.

## Clinical characterization of TTS

An overview of the clinical and diagnostic features of VITT in the UK was presented. VITT was described as a cause of TTS but not synonymous with it. As cases were collected from daily clinical meetings during the vaccination campaign, a pattern consisting of five equally weighted clinical features emerged, and by using those the likelihood of the case being VITT could be categorized into definite, probable, possible, and unlikely^18^. The initial female over-representation of TTS cases appeared to reflect the demographics during the early rollout of the adenovirus-vector vaccine in the UK. Of note, the administration of second doses of COVID-19 vaccine in 40 UK patients who had either definite (26), probable (2) and possible (12) VITT after a first dose of Vaxzevria did not lead to recurrent VITT^19^, including in the few subjects (*n* = 5) who had received a second dose of Vaxzevria.

This was corroborated by the work conducted in an independent research group in Germany. The latter demonstrated that anti-PF4-antibodies in TTS vaccinees are transient^20^. The detection of anti-PF4 antibodies is a crucial marker for VITT but available assays have different sensitivities^21^, and ELISA based approaches can be combined with specific functional assays. It has been previously shown that anti-PF4 antibodies associated with VITT do not cross react with the spike protein, indicating that VITT is specifically induced post adenovirus vector vaccination^22^. The human in vivo evidence that the anti-PF4 response in VITT is neither related to the spike protein nor to SARS-CoV2 came from data collected in a cohort of 11 patients with a history of VITT and subsequent COVID-19. In these patients no significant increase in anti-PF4 antibody levels was observed after recovery from COVID-19^23^.

As VITT occurs starting from 5 days post-vaccine administration, the anti-PF4 B cell response does not align with the notions of the conventional immunological response post primary antigen exposure and appears to be a secondary immune response. Recently, the molecular signature of clonotypic anti-PF4 antibodies was identified in five patients, defined as a single IgG heavy (H)-chain species paired with a single lambda light (L)-chain species, and all L-chains were encoded by the identical IGLV3-21*02 gene subfamily^24^. These results may reveal a shared pathway of antibody production in VITT patients and could point to a possible genetic predisposition at the basis of the syndrome.

According to one working hypothesis, complexes formed by PF4 and adenovirus vector vaccines together with vaccination induced strong immune activation could lead to the formation of anti-PF4 pathogenic autoantibodies triggering platelet activation and the downstream prothrombotic cascade^25^. Adenovirus vaccine constituents binding to PF4 may induce conformational changes in PF4 and create potential neoantigen(s), responsible for marginal zone B cells activation. This latter immunobiological process still requires further investigation. However, data generated with super-resolution microscopy show that vaccine components form complexes with PF4 to which anti-PF4 antibodies obtained from VITT patients bind in vitro. The participants reflected on the fact that anti-PF4 antibodies can be found in ~5% of the population^26,27^, but these common antibodies do not activate platelets and are likely of no or only minor clinical relevance. In very rare cases pathogenic, platelet activating anti-PF4 antibodies can also occur independent of COVID-19 vaccination and heparin-induced thrombocytopenia (HIT) syndrome, as occurred post viral infections or knee replacement surgery^28^. HIT is an adverse reaction to the drug heparin.

An overview of the clinical cases of vaccine associated TTS post Vaxzevria in Australia was provided. These cases in Australia are classified according to the International Network of Special Immunization Services approach to characterize risk factors and mechanisms underlying adverse events of special interests following vaccination^29^. Of relevance, the mortality cases post second dose of Vaxzevria in Australia appeared to be associated with a shorter dosing interval than the current national recommendations. Furthermore, ongoing research plans to study genetic risk factors and the characterization of B-cell clones producing autoantibodies in VITT via multi-omics were discussed among the participants.

Interestingly, data generated by mass-spectrometry showed that anti-PF4 antibodies obtained from VITT patients are monoclonal or oligoclonal, in contrast to anti-PF4 antibodies in conventional HIT, which are always polyclonal^30^. It was commented that beside the dichotomous distinction of monoclonal versus polyclonal humoral response between VITT and HIT, it will be crucial to understand the exact binding epitopes to PF4 shared or not between the two antibody responses. Recent data suggests that antibody binding to un-complexed PF4 seems to be the key differentiating feature of VITT from HIT and spontaneous HIT cases^31^, however the degree of specificity of this assay needs to be evaluated in larger studies. VITT antibodies only occasionally activate platelets in the presence of heparin as in the serotonin release assay, but consistently activate PF4-treated platelets, in line with previous findings. Thus, it is important to only use PF4-treated platelets in functional testing for VITT.

The attendees discussed the relative merits of centralized versus decentralized laboratories for functional testing to reliably detect TTS and VITT cases worldwide. Also, while VITT is associated with high optical density (OD) of anti-PF4 antibodies in PF4-polyanion ELISAs (HIT ELISAs) there are exceptions, and many HIT antibodies also have high ODs in this assay. In addition, a small percentage of healthy individuals are positive in this ELISA (typically with low ODs). Due to the short time frame for detection of the anti-PF4 antibodies in sera (sometimes already 5 days post-immunization), the most likely immunological explanation is that TTS vaccinees harbored pre-existing anti-PF4 B cell clones which become activated post-vaccination. The mono versus the poly-clonality antibody concept, as well as the type and signature of B cell population involved in the immune response will require further study.

## Understanding the biological mechanisms and impact of vaccine composition on TTS

Scientific evidence emerged aiming at unraveling the biological mechanism and the structural interactions between the adenovector and PF4.

AstraZeneca presented the company’s position on the hypothesized mechanism leading to TTS. In their view ChAdOx1-PF4 complexes are recognized by pre-existing B-cell clones encoding pathogenic anti-PF4 antibodies (IgG). The boosted production of TTS inducing anti-PF4 IgG subsequently triggers thrombosis via activation of platelets and neutrophils. The presence of low level anti-PF4 antibodies and platelet activation does not predict the induction of TTS. Indeed 1–2% of individuals are positive for low levels of anti-PF4 IgG and these individuals do not develop TTS post–vaccination^32,33^. Also, vaccination with Vaxzevria does not generally increase anti-PF4 levels in healthy vaccine recipients who did not develop VITT^32^. According to the company’s working hypothesis, the nature of the individual host response determines whether TTS is induced upon exposure to the ChAdOx1-PF4 complex. This is in line with the in vitro binding data between PF4 and ChAdOx1 reported by Baker et al.^34^. The anti-PF4 IgG will then lead to the downstream activation cascade as it has been shown that IgG from TTS patients is capable of activating platelets and neutrophils and inducing thrombosis in a murine model (hFcγRIIa/hPF4)^35^.

Two academic research groups studied the interactions between the adenovirus capsid and PF4 and demonstrated that the capsids of the adenoviruses Ad5, ChAdOx1 and Ad26 can form a complex with PF4^34^. It was shown in ChAdOx1 that this complex is sufficiently stable to support tertiary complex formation with anti-PF4 IgG. Computational modeling and Brownian dynamic simulations, utilizing the experimentally determined capsid structure indicated that the interactions are not randomly distributed over the virion surface but mainly occur at the interfaces between hexons. Data was described in which purified hexons, with no DNA component, were also able to interact with PF4 in surface plasmon resonance studies. Also, research focused on the molecular mapping of the PF4–ChAdOx1 adenovector interaction and on the biochemical characterization of VITT antibodies confirmed the binding data from Baker et al. It was demonstrated that PF4 binds to ChAdOx1 by biolayer interferometry using both streptavidin and amine-coupling sensors. A mutagenesis study allowed the identification of the PF4 amino acid residues involved in ChAdOx1 binding. It appeared that the ChAdOx1 binding site on PF4 overlaps with the heparin binding site but not all amino acids are shared between the two binding sites^36^. In addition, the binding of anti-PF4 antibodies from VITT patients were restricted to eight surface amino acids on PF4, all of which located within the heparin-binding site. It was also reported that some VITT patients had anti-PF4 antibodies binding only to one site on PF4 (the heparin binding site), while other VITT patients were able to bind to two distinct sites on PF4 (a characteristic shared with antibodies from some HIT patients). Taken together, the induction of disease specific anti-PF4 antibodies in VITT and HIT result in similar but yet distinct molecular interactions that are reflected by the apparent phenotypical similarity of the two diseases.

Janssen presented another hypothesis for the pathogenic mechanism at the basis of TTS. According to this, TTS is not mediated by the adenovector capsid interacting with PF4. The company presented the results of several experiments deploying a range of methodologies including surface plasmon resonance, bio-layer interferometry and dynamic light scattering to study the interaction between PF4 and Ad26.COV2.S in vitro. No strong binding of Ad26.COV2.S and PF4 could be demonstrated in any of these experiments. The company suggests that the contrasting results obtained by Baker et al.^34^ could be due to an experimental artefact caused by NaOH treatment, which is used to regenerate the chip. Instead of a direct role for the adenoviral capsid of Ad26.COV2.S in TTS, Janssen hypothesizes that adenovirus vector vaccines induce TTS by a combination of the SARS-COV-2 spike protein, the vaccine induced inflammatory milieu and a specific predisposition of the patient.

Both Janssen and AstraZeneca will continue research into the identification of risk factors for TTS following adenovirus vector vaccinations.

In general, it was commented that better characterizing the commonalities and differences between the HIT and VITT antibodies will help us to understand why the un-complexed PF4 can react better than PF4-heparin in the serotonin release assay used to assess platelet functionality and further elucidate the basis of the very restricted nature (mono-/oligo-clonality) of the VITT antibody specificity. Accordingly, PF4 could be stabilized on the viral capsid and bind to it, which would allow the B cell receptor to activate the B-cell. These B-cells then produce anti-PF4 antibodies, which form immune-complexes after binding to PF4 and trigger platelet activation with the FcyRIIa part of the anti-PF4 antibodies, leading to thrombocytopenia and contributing to thrombosis.

It was originally hypothesized that Vaxzevria and JCOVDEN associated impurities may drive the immune thrombotic activation cascade. However, given the remarkable differences between the two vaccines in terms of impurities due to the differences in manufacturing of the vaccines^37^, this is rather unlikely. Of note, the contribution of vaccine components could still have an adjuvant effect, although there is currently no experimental data in support of this possibility.

## Overall conclusions and next steps

Real world data, based on the use of electronic medical records and health claims, has proved useful to identify TTS amongst vaccinated subjects, and specifically VITT in Europe, North America and Australia, while only 3.2% of these cases have been notified in LMIC settings. It can be speculated that low reporting of VITT in the LMICs may be partially related to the scarce availability of anti-PF4 antibody tests in these regions, as well as to underreporting of very rare adverse events and differences in the pharmacovigilance system across the globe. Therefore, the group highlighted the need to improve surveillance activities especially in LMICs and to contribute to the creation of testing and surveillance networks. In this regard, countries where adenovirus vector vaccines are widely administered, such as in India and Mexico, have no report of VITT but also likely limited facilities and resources to diagnose it.

Of note, the recent work performed by Tom Gordon^24^ unveiling the molecular signature of stereotyped clonotypic anti-PF4 antibodies in five VITT patients supports the hypothesis that VITT may develop in patients with a defined genetic background. The ethnicity component was deemed a key element to be further explored. Ongoing host genome sequencing analyses are being carried out in Europe and Australia to identify any potential genetic risk factors involved in the etiology of VITT, which may also contribute, to some extent, to the lower incidence of TTS and VITT in Asia and South America.

**Fig. 1:**
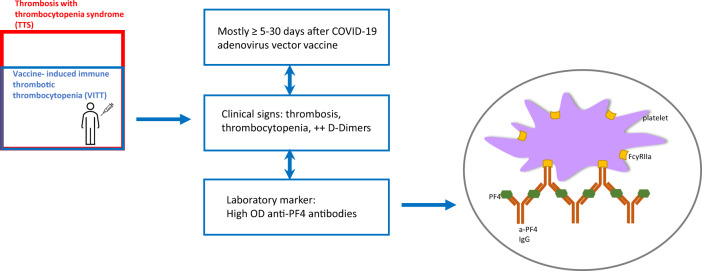
VITT: an overview of the disease. VITT is a newly identified disease, which occurs very rarely post adenovirus vector COVID-19 vaccines. Crucial clinical and laboratory signs of the disease are: thrombosis with thrombocytopenia, elevated D-dimers, high levels of anti-PF4 antibodies as measured by ELISA. Secreted anti-PF4 immunoglobulins (IgG) can form aggregates with PF4 in the blood circulation. These aggregates can activate platelets and trigger the downstream prothrombotic cascade. Worldwide, the exact number of VITT among all TTS cases after adenovirus vector COVID-19 vaccination remains currently unresolved due to the limited availability of anti-PF4 antibody testing.

The experts collectively emphasized that the presence of antibodies to platelet factor 4 is a key feature and driver of VITT with an adenovirus vector COVID-19 vaccine, which must be considered a specific newly identified syndrome (Fig. 1). Background TTS cases in the general population do exist but have a different pathophysiology; these very rare cases are not associated with vaccination and should not be confused with the specific VITT cases. Additional research is needed to discern the various forms of TTS, and to understand to what extent these are related to vaccination.

Regarding the role of the spike protein in platelet activation and VITT, according to the latest results published^23^, the spike protein present in the COVID-19 vaccines is not considered to be the main driver of the immune response at the start of the prothrombotic cascade as VITT patients experiencing COVID-19 after vaccination do not show a boosted anti-PF4 antibody response nor a relapse of VITT. These data do not exclude a potential role of the spike protein in the co-inflammatory response and, in this regard, characterization of (co-)factors contributing to or associated with the pro-inflammatory milieu potentially involved in triggering VITT remains one of the top priority areas requiring further research.

The current research on the interaction between PF4 and the adenovector from Vaxzevria and JCOVDEN was discussed extensively. Most results point towards a model in which binding of the capsid protein of both adenovectors, ChAdOx1 and Ad26, to PF4 results in complex formation and subsequent downstream activation of PF4-specific B-cells. The produced anti-PF4 antibodies then cause prothrombotic activation of several cells in the blood. Upcoming research data should shed light on some key and yet unanswered questions, including to what extent the shape complementarity and electrostatic mechanism contribute to this interaction; whether the frequency of interaction is relevant to the mechanism of VITT in a biological context above a certain threshold and which tertiary partner or partners are affecting the complex formation. On this latter aspect, despite initial attempts^38^, investigators have yet to find a common component associated with the two adenovirus vector vaccines contributing to the complex formation. By comparing the results obtained from multiple studies using different methodologies, the experts noted that despite the tremendous amount of scientific research done so far, several areas require additional investigation. This includes understanding how PF4 binding occurs in vivo across different adenoviral vaccine platforms, and further in-depth characterization of the immune responses mounted by VITT subjects. As VITT is most likely a multifactorial disease, determining all the factors and their relative contribution to the pathophysiology of VITT is crucial to minimize its incidence and define the future application of adenovirus vector vaccine technology.

## Declarations

The views, findings and conclusions expressed in this article are the personal views of the authors and may not be understood or quoted as being made on behalf of or reflecting the position of the EMA or its committees or the organizations with which the authors are affiliated, including the Centers for Disease Control and Prevention.
